# The economic value of the Brazilian Amazon rainforest ecosystem services: A meta-analysis of the Brazilian literature

**DOI:** 10.1371/journal.pone.0268425

**Published:** 2022-05-19

**Authors:** Roy Brouwer, Rute Pinto, Anders Dugstad, Ståle Navrud

**Affiliations:** 1 Department of Economics and the Water Institute, University of Waterloo, Waterloo, Canada; 2 Institute for Environmental Studies, Vrije Universiteit Amsterdam, Amsterdam, The Netherlands; 3 Ecohydrology Research Group, Department of Earth and Environmental Sciences, University of Waterloo, Waterloo, Canada; 4 School of Economics and Business, Norwegian University of Life Sciences, Ås, Norway; North Carolina State University, UNITED STATES

## Abstract

The main objective of this study is to assess the economic value of the Brazilian Amazon’s ecosystem services accruing to Brazilians based on a meta-analysis of the Brazilian valuation literature. Insight in these local values provides an important benchmark to demonstrate the importance of preserving the Brazilian Amazon forest. The review covers almost 30 years of Brazilian valuation research on the Amazon, published predominantly in Portuguese, highlighting a high degree of study and data heterogeneity. The estimated mean value of the provision of habitat for species, carbon sequestration, water regulation, recreation and ecotourism to local populations is about 410 USD/ha/year. The standard deviation is however high, reflecting a wide dispersion in the distribution of values. Between 50 and 70 percent of the variation in these values can be explained with the help of the estimated meta-regression models, resulting in considerable prediction errors when applying a within-sample resampling procedure. These findings demonstrate the need for a more robust, common ecosystem services accounting and valuation framework before these values can be scaled up and aggregated across the entire Brazilian Amazon.

## 1. Introduction

The valuation of ecosystem services (ES) is subject of growing enthusiasm and academic debate as the number of valuation studies and meta-analyses continue to grow, in particular for terrestrial ecosystems such as forests. Economic valuation of ES helps to make the values of nature more visible, as advocated in global initiatives like The Economics of Ecosystem Services and Biodiversity (TEEB) and the World Bank-led global partnership on Wealth Accounting and Valuation of Ecosystem Services (WAVES). Tropical rainforests are among the most valued and threatened ecosystems in the world. Although several studies synthesize the results from forest valuation studies worldwide [e.g. 1–4], none of these studies investigate the economic values of the ES delivered specifically by the Amazon, the largest tropical rainforest in the world, covering some 5.2 million km^2^ across eight South American countries.

The main objective of this study is to assess the economic values of ES provided by the Amazon rainforest in Brazil to local populations, in particular regulating services such as carbon sequestration and water regulation, and cultural ecosystem services such as recreation and eco-tourism, and habitat for species. Insight in these local values is paramount for comparison with the opportunity costs of forest preservation in cost-benefit analysis [3, 5, 6]. These opportunity cost estimates are typically based on market values of alternative land uses in the Amazon, such as agriculture and exploitation of the provisioning services of the Amazon rainforest [7–11]. The number of studies assessing the non-market ES to support decision-making towards sustainable Amazon forest management is limited. Most economic valuation studies related to the Amazon focus on provisioning services such as timber and non-timber forest products, using market prices to estimate the benefits of avoided deforestation and forest degradation [e.g. 12–16]. In this study, the existing Brazilian valuation literature addressing regulating and cultural ES is synthesized in a meta-analysis following best practice guidelines [e.g. 17]. A meta-regression model is estimated to identify which study characteristics explain the variation observed in the empirical literature, and assess to what extent the underlying value function can be used to scale up the local valuation studies to the Brazilian Amazon as a whole for spatial targeting purposes and inform national ES conservation efforts.

Although the Amazon rainforest and its ES transcend national boundaries [e.g. 18, 19], the scope of the study is limited to Brazil where most of the rainforest (60%) and most of the valuation studies are found. The majority of these valuation studies are published in Portuguese and therefore not easy accessible internationally. A limited number of valuation studies exist outside Brazil, asking in particular European citizens [20–22] for their willingness to pay (WTP) to conserve the Amazon rainforest or asking European valuation experts for their assessment of the economic value of the Amazon rainforest based on their expert judgment [23, 24]. The novelty and value added of the meta-analysis presented here is that it focuses on local values of ES provided by the Brazilian Amazon to Brazilians reported in the Brazilian literature.

## 2. Data collection

Relevant Brazilian valuation studies were identified using search strings in English and Portuguese in international databases such as Science Direct and Scopus, national databases such as RCIPEA, BDTD and SCIELO, sites specifically related to the conservation of the Brazilian Amazon such as imazon.org.br, and university thesis repositories such as http://bdtd.ibict.br/. Search strings included, among others, combinations of the words ‘Amazon’, ‘ecosystem services’, ‘benefits’, ‘economic valuation’, ‘non-market valuation’, ‘stated preferences’, ‘revealed preferences’, ‘contingent valuation’, ‘choice experiment’, ‘travel cost’, ‘payments for ecosystem services’, ‘carbon sequestration’, ‘recreation’, ‘ecotourism’, ‘water cycle’, ‘hydrological services’, ‘cultural ecosystem services’ and ‘regulating ecosystem services’.

**Fig 1:**
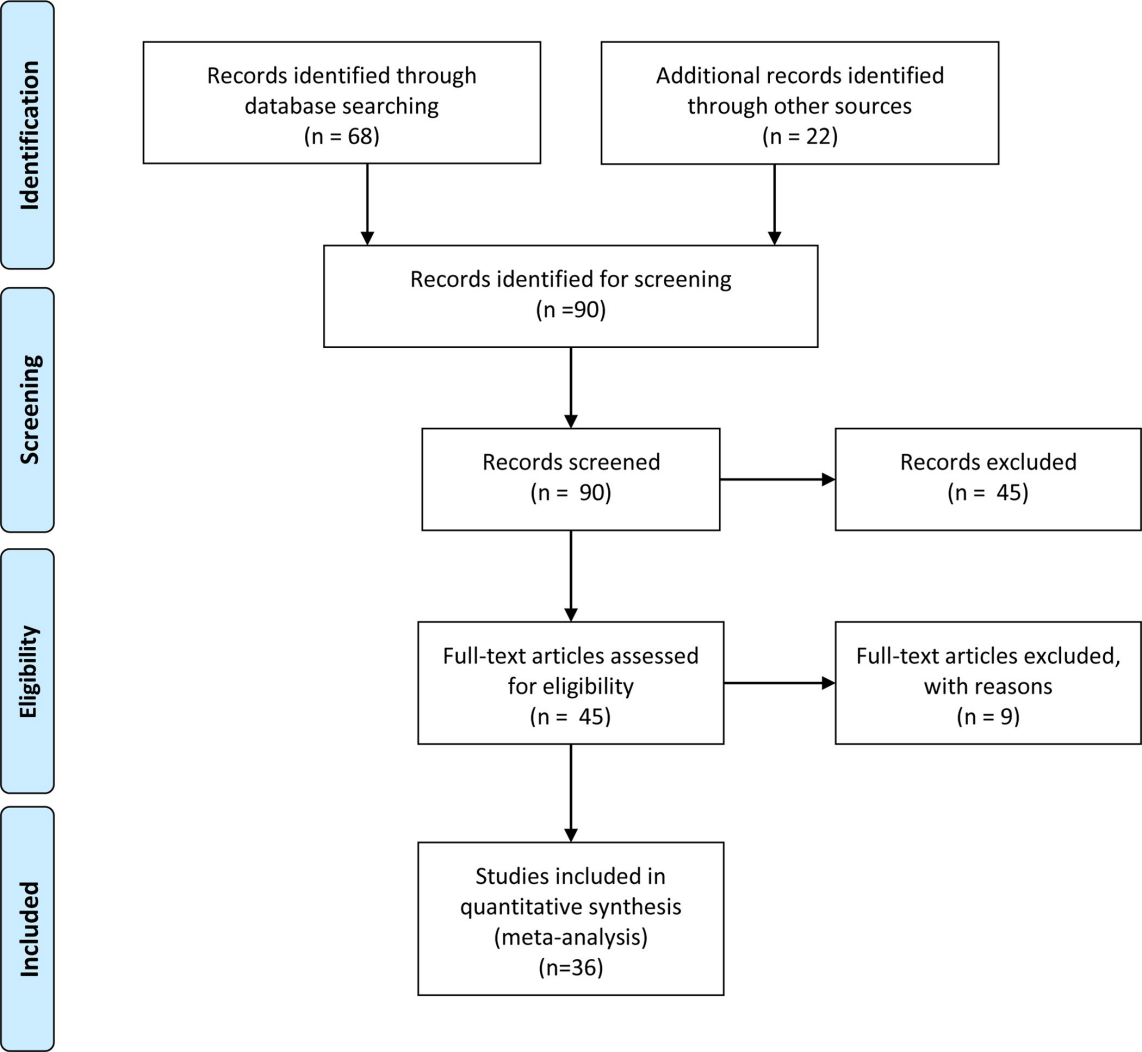
PRISMA flowchart.

The initial data search yielded 90 studies that presented a wide variety of information regarding the benefits provided by the Brazilian Amazon forest (Fig 1). A first inspection of this initial set of studies resulted in the exclusion of studies that estimate the opportunity costs of provisioning services using market prices, such as specific wood trees, fruits and seeds sold in local markets by small communities [e.g. 15, 25]. This is half of the initial number of studies. These studies were excluded, because the forest products could in most cases not be traced back to the specific area of origin or the values of these products were measured in incomparable units (e.g. per kg). Also excluded are studies examining the value of grasslands in the Amazon used for cattle grazing. The latter represent the opportunity costs of Amazon rainforest conservation. In a second, more detailed cross-check of the remaining studies, another 9 studies were excluded because the same results were either presented twice in different publications or the studies borrowed estimates from other studies that were already included in the database [e.g. 26]. The latter includes, for example, the values paid for ES under the Bolsa Floresta Payments for Ecosystem Services (PES) program [e.g. 27, 28].

Although the level of detail about the primary valuation studies varied, study quality was not applied as a selection criterion. This is typically considered a major advantage of meta-analysis in that it does not prejudge research findings based on the original study’s quality [29]. Moreover, although various state-of-the-art guidelines have been published for stated preference research [e.g. 30], there do not exist clear-cut objective benchmarks to identify poor quality valuation studies for either market or non-market valuation of ES. Hence, some degree of subjective judgment when screening the studies’ quality would be inevitable and defeat the purpose of conducting the meta-analysis. The study presented here aims to take stock of the Brazilian valuation literature related to the Brazilian Amazon and synthesize the values that have been estimated in the existing literature for regulating and cultural ES provided by the Brazilian Amazon rainforest to local populations.

After this screening, 36 studies remained, generating 61 point estimates. The list of studies included in the database and their bibliographic details are presented in S1 Table in S1 File to this paper. The studies cover 27 years of Amazon valuation research (1990–2017). Most studies (58%) produced 1 estimate, while one study generated as many as 10 observations (see S2 Table in S1 File). Multiple values from single studies were extracted if their differences could be explained with the help of the explanatory factors included in the meta-regression models. On average, a study generated 1.7 value estimates. Half of the studies were published as peer reviewed articles, a quarter as non-peer reviewed papers like conference papers, and 17 percent as academic thesis. Two studies were published as reports and one as a book chapter. Except for [31], all studies were published in Portuguese. For one study we were also able to find a corresponding English article published in the international literature [32].

All value estimates were translated into 2020 USD/ha/year using the OECD’s Purchasing Power Parity (PPP) adjusted exchange rate and the World Bank’s Consumer Price Index (CPI) for Brazil. Values were made comparable over time by expressing them in 2020 price levels in Brazil (using the CPI), and subsequently converting them into US dollars (using the PPP). Where possible, the aggregated values across the population of beneficiaries reported in the publication were used and divided by the size of the area delivering the ES. If studies did not detail the size of the area for which the values were estimated, this information was looked up in other secondary data sources. The original values extracted from the individual valuation studies and their measurement units, the population of beneficiaries and the area size are presented in S3 Table in S1 File.

**Fig 2:**
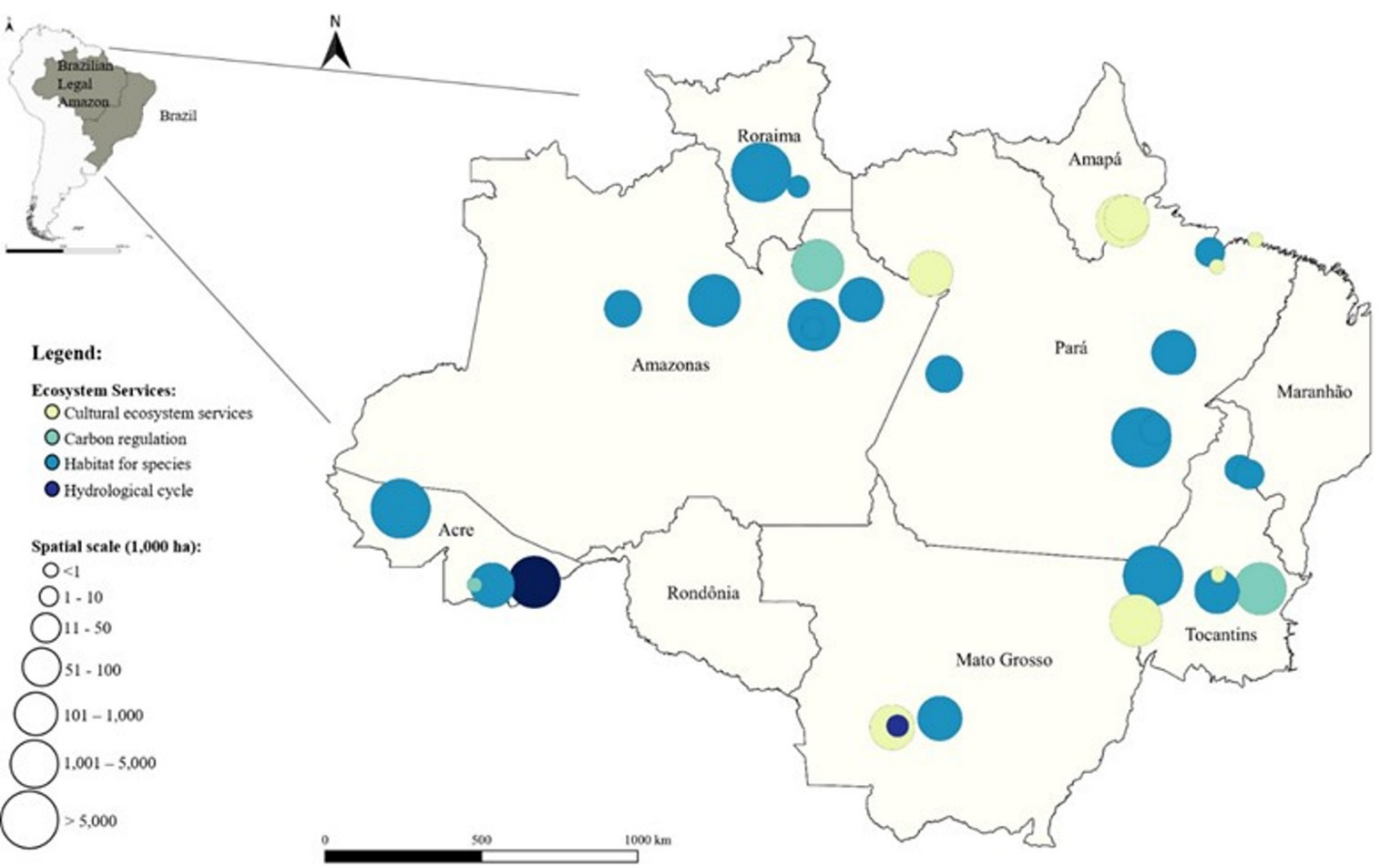
Spatial distribution of Brazilian valuation studies included in the database and their scope and scale of analysis. Note: the map excludes the valuation studies that aimed to value the whole Brazilian Amazon. Sources: The maps underlying Fig 2 are based on open access files obtained from the Brazilian Institute of Geography and Statistics (Instituto Brasileiro de Geografia e Estatística–IBGE).

Studies were carried out in different parts of the Legal Brazilian Amazon rainforest. This is illustrated in Fig 2. Most value estimates originate from the states Amazonas (27%) and Pará (22%), followed by Tocantins (17%). Tocantins falls only partly in the Amazon biome. The number of observations where the Brazilian Amazon was valued as a whole account for 8 percent of all the values. The rest is divided across 6 other states (see also S1 Fig in S1 File). The states Maranhão bordering the Atlantic Ocean and Rondônia bordering Bolivia in the west are geographically speaking underrepresented.

More than a third of the studies (36%) investigated the benefits of conserving protected areas, such as extractive reserves (RESEX) or national forests [e.g. 33–35]. Almost 30 percent of the studies considered environmental degradation the main threat to the conservation of the Amazon rainforest [e.g. 36], while 22 percent of the studies evaluate the impacts of deforestation on the provision of ES [e.g. 37, 38], followed by 19 percent that focus on river pollution and overextraction of water [e.g. 39, 40]. Other threats considered in the studies are related to cattle production expansion [41], tourism and urban expansion [42, 43], mining [31, 35], or threats related to the Amazon’s water cycle, such as the construction of hydroelectric dams [44, 45]. Wildfires were addressed in three studies [38, 46, 47]. Fires are used in the Amazon to clear areas for agriculture or pasture activities [e.g. 48, 49], increasing the risk of spreading fires to other areas of the forest.

In order to control for location and population characteristics in the study areas, secondary data were collected from different sources, most importantly the Brazilian Institute of Geography and Statistics (IBGE). An overview of the collected socio-economic, land use pressure and environmental indicators is presented in S4 Table in S1 File. This secondary data was included in the meta-analysis at the lowest spatial aggregation level possible, i.e. at municipality level, and related to the specific study year of the valuation study or closest to the study year if the secondary data happened to be unavailable for a specific study year.

## 3. Meta-regression model structure

The meta-regression models presented here aim to explain the variation found in existing Brazilian valuation studies of the Amazon forest’s ES. Although the study zooms in on specific ES (regulating and cultural ES), a large amount of data heterogeneity is expected given the fact that valuation studies apply different valuation methods, ranging from market prices to non-market valuation procedures such as stated and revealed preferences methods, in different Brazilian states across different years. This data heterogeneity is partly captured in the deterministic part of the meta-regression models through the inclusion of control factors, but part is also expected to end up in the error terms. These error terms are not a priori expected to be identical and independently distributed (iid) across the data points, not least because many studies included in the meta-analysis produce multiple correlated estimates, for example due to the use of the same research design or drawing upon the same population sample. This data heterogeneity, differences in effect size variance and non-independence of observations must be accounted for in meta-regression modelling [17]. To this end, the meta-regression model is specified as a generalized least squares (GLS) model:

$$y_{ij}=x_{ij}^{\prime }\beta {+\varepsilon }_{ij}\;with\;\varepsilon_{ij}∼[0,\sigma_{j}^{2}]$$ (1)

where *y*_*ij*_ denotes the dependent variable, i.e. the value estimate *i* for the ES provided by the Amazon forest in Brazil derived from study *j*, *x*_*ij*_ is a vector of regressors, and *β* is a vector of associated coefficients. GLS regression is able to account for both random and fixed variation in the values stored in the database, in particular multiple observations from single studies, which may lead to heteroskedasticity or correlated error terms. Fixed-effects describe the effects of the regressors *x*_*ij*_ on the dependent variable *y*_*ij*_ for the sample as a whole, while random-effects may vary across studies and capture possible heterogeneity between studies. The error term *ε*_*ij*_ denotes the standard error for each observation *i* which may vary across *j* studies and is assumed to be distributed independently of the regressors.

The regressors *x*_*ij*_ in Eq (1) capture different groups of influencing factors, related to the characteristics of (i) the valued ES $x_{ij}^{ES}$, (ii) the areas where these ES are provided, e.g. whether or not the areas are protected $x_{ij}^{LOC}$, (iii) the population of beneficiaries $x_{ij}^{POP}$, (iv) the methods applied to value the ES $x_{ij}^{METH}$, and (v) their publication $x_{ij}^{PUB}$:

$$y_{ij}=x_{ij}^{\prime }\beta {+\varepsilon }_{ij}=x_{ij}^{ES}+x_{ij}^{LOC}+x_{ij}^{POP}+x_{ij}^{METH}+x_{ij}^{PUB}{+\varepsilon }_{ij}$$ (2)

Besides extracting data and information from the identified publications in which the studies are presented, external data sources (see S4 Table in S1 File) were consulted to capture as much of the observed heterogeneity in the value estimates as possible. The models presented in this paper are estimated in Stata version 16.1.

## 4. Results

### 4.1. Database description

The TEEB [50] framework is used to define the various categories of ES. This framework is closely related to the Common International Classification of Ecosystem Services (CICES) [51]. Just over 15 percent of all value estimates relate to the Brazilian Amazon’s regulating services, of which most (60%) refer to carbon storage and sequestration and 40 percent to the water cycle. These regulating ES benefit Brazilians directly and indirectly, for example by reducing local or regional greenhouse effects and sustaining water supply and water filtration. Another 21 percent of the values refer to cultural ecosystem services, mainly recreational activities such as bathing and fishing, and ecotourism. More than 60 percent of all the values (62%) relate to habitat for species and mainly measure the Brazilian Amazon ecosystem’s non-use value. These non-use values are classified as ‘habitat for species’ in the TEEB framework and as ‘cultural ecosystem services with nonuse value’ in CICES. As expected, these values are in most cases elicited using stated preference methods like contingent valuation (CV) and choice experiments (CE). A very limited number of studies [37] also focus on the forest’s ability to maintain genetic diversity. These estimates refer to future possible use values (called ‘option values’ in the environmental economics literature) and are excluded from the analysis here because the reported value in the literature was based on another study that was not related to the Brazilian Amazon.

The size of the areas to which the ES relate is in most studies very large. Exceptions are [52] who focus on a natural beach in Tocantins and [53] who examines the implementation of the Bolsa Floresta program among 272 families in a local municipality in Amazonas, while [54] refer to a relatively small reserve called Reserva Extrativista Chico Mendes in Acre, where sustainable extraction of resources is only allowed under strict conditions. Excluding observations that relate to the whole Amazon rainforest, the mean area size is just over 40 thousand square kilometers and the median 400 square kilometers of forested land. Aggregated, the areas together cover approximately 27 percent of the whole Brazilian Amazon. Close to 40 percent of the observations are found in protected areas. The size of these protected areas varies considerably from 1.2 hectares to 895 thousand square kilometers, with a mean of 40 thousand square kilometers.

Although the studies cover more than a quarter of the Brazilian Amazon, it is important to point out that the majority of the studies estimated local values held by local populations. More than half of the studies (53%) were conducted in cities and municipalities in the Brazilian Amazon. This includes five studies in the capital cities of the states of Amazonas (Manaus) and Acre (Rio Branco). Four studies interviewed local residents living either in a reserve, national park or remote rural area close to a protected area, while two studies focused on the importance of the Amazon river system for local fishermen [45, 55]. Only 6 studies estimated values at the scale of the entire Amazon or an entire state (e.g. Tocantins). None of the studies estimated the possible values held by residents living in the largest cities in Brazil like São Paulo, Rio de Janeiro, Salvador, Fortaleza, Belo Horizonte or Recife. Only one study included a sub-sample of residents from Brasilia [31], the capital of Brazil, and elicited their nonuse values for the preservation of the Amazon.

Due to the relatively small area sizes in some studies, this resulted in a few very high per hectare values for cultural ES, including habitat for species, estimated with the help of stated and revealed preference techniques. Eight outliers were identified, five based on CV, two travel cost (TC) estimates and one based on market prices, by comparing their values to the mean value derived from the same valuation method. This is illustrated in S2 Fig in S1 File. These very high values (>5,000/ha/year), are orders of magnitude higher than the average values obtained using the same valuation methods and were therefore removed from the univariate analysis presented in the next section.

### 4.2. Economic value estimates

The remaining 53 value estimates were, amongst others, grouped according to the ES and valuation methods to which they pertain in Table 1. The overall average value estimate for the Amazon ES in Brazil based on these 53 observations is 411 USD/ha/year with a standard error of 123. This is higher than the average value reported in [3] for the aggregate economic value of the provisioning, regulating and cultural ES provided by tropical forests worldwide (266 International $/ha/year in 2012 prices) based on 53 observations from the TEEB database, including 5 value estimates from Brazil. However, when updating the mean value reported in [3] to 2020 prices using the World Bank’s world CPI, the aggregate economic value increases to 524 I$/ha/year (there exists no difference between international and US dollars [56]). Our average estimate for regulating and cultural ES based on 53 Brazilian Amazon rainforest values is 22 percent lower than this value, while also the variation coefficient in the meta-analysis presented here is 30 percent lower than the variation coefficient in [3], signaling a narrower distribution of value estimates based on Brazilian valuation studies only. The range of values in the database is nevertheless relatively wide, as can also be observed from the 95 percent confidence interval around the mean value estimate between 165 and 658 USD/ha/year.

**Table 1:** Average economic values (2020 USD/ha/year) for the Brazilian Amazon across ecosystem services and valuation methods.

	Mean value^1^	95% confidence interval	Min-max value	*N* ^2^
Aggregate value for all ecosystem services	411.2 (122.7)	165.0–657.5	0.04–4,354.6	53
**Ecosystem service**				
Carbon regulation	333.3 (144.3)	-37.7–704.3	59.2–1,032.7	6
Water cycle	150.8 (74.6)	-86.6–388.2	2.1–352.9	4
Recreation and ecotourism	410.3 (328.1)	-365.6–1,186.3	0.06–2,649.6	8
Habitat for species	454.6 (170.1)	109.0–800.2	0.04–4,354.6	35
**Valuation method**				
Contingent valuation	627.5 (213.1)	191.1–1,064.0	0.04–4,354.6	29
Choice experiment	56.5 (11.8)	5.7–107.2	40.6–79.5	3
Travel costs	2.1 (1.1)	-11.9–16.1	0.9–3.2	2
Payments for ES	112.8 (90.3)	-1,034.9–1,260.5	22.5–203.1	2
Market prices	124.5 (63.7)	-13.2–262.1	0.05–871.2	14
Avoided damage costs	485.2 (282.6)	-730.8–1,701.3	89.6–1,032.7	3

^1^ Standard error between brackets.

^2^ Number of observations.

The same applies to the 95 percent confidence intervals for the different ES and their valuation methods in Table 1. Many of the 95 percent confidence intervals around the mean economic values for the four ES and six valuation methods include a negative lower bound. This wide spread in values undermines their use and usefulness when aiming to transfer them to other parts of the Brazilian Amazon where no valuation studies were conducted to inform forest conservation policy about the importance of the various ES involved.

Habitat for species has the highest average economic value based on the largest number of observations and is significantly higher than the regulating services for carbon and water (based on the Mann-Whitney (MW) test), but not for recreation and ecotourism (MW Z = -0.468; *p*<0.657). This suggests that the Brazilian citizens surveyed in the studies in the database attach a relatively high value to the protection of the Brazilian Amazon ecosystem for non-use reasons, although this value is only 11 percent higher than the recreational value of the Amazon. Seven of the 36 studies (19%) estimated the recreational value of the Brazilian Amazon by investigating the travel costs and revenues from visitors to national forests, parks, lakes and rivers, while local ecotourism is part of the total economic value estimated, for example, for protected areas in [57]. Remarkable is the very high standard error for this cultural ES, suggesting that the recreational and ecotourism values differ substantially across the Brazilian Amazon.

Within the wildlife habitat category, a wide variety of valuation studies exists, most of which estimate public WTP to preserve the Brazilian forest ecosystem [e.g. 31, 58], or ask for the public’s willingness to accept (WTA) compensation for the loss of natural habitats and resources [e.g. 53, 59]. Around half of these studies examined forest preservation in general, considering different threats such as mining [3] or subsistence agriculture [53, 60], or focus on the existence value of protected areas [33, 34]. [35] looked specifically at the existence value of the ‘canga’ ecosystem in the Brazilian Amazon, an ecosystem type that is known for hosting rare endemic vegetation as well as scenic landscapes. Close to a third of the studies in this category furthermore estimate the existence value of river ecosystems or natural beaches [e.g. 44, 59, 61]. This includes the restoration benefits of streams [62] or preventing negative impacts from threats like oil spills [63]. The remaining studies look at the preservation of natural resources in general, for example by considering the natural area surrounding a harbour [58] or estimating the value of a state’s natural capital including a pool of different types of ecosystems (tropical forest, swamps, bush areas) [64].

Although the mean value for carbon sequestration is twice as high as for water regulation, the difference for these two regulating services is statistically not significantly different due to the relatively high standard errors and low number of observations (MW Z = -1.069; *p*<0.352). Two studies [46, 65] link carbon sequestration in the Brazilian Amazon to the international carbon market, originally set up under the UN’s 1997 Kyoto protocol on climate change, while it is linked to a PES scheme in Amazonas in [66]. Although it has been argued that PES schemes in Brazil offer an interesting opportunity to implement other related programs such as Reducing Emissions from Deforestation and Forest Degradation (REDD) [67], and Brazil is considered a frontrunner in REDD+ planning [68], none of the valuation studies in this meta-analysis are linked to a local or regional REDD or REDD+ project in the Brazilian Amazon.

Turning to the valuation methods in Table 1, the CV method produces, on average, the highest values based on the largest number of observations, mainly related to the protection of habitat for species. This is expected due to the fact that these values include, theoretically, both use and non-use values, and possibly also because of a positive hypothetical bias associated with stated preferences research that might lead to higher values [e.g. 69, 70]. The two TC studies included in Table 1 [71, 72] generate the lowest values for the recreational services provided by the Brazilian Amazon, such as visiting a reserve or national park.

Seven of the 29 CV-based values (24%) relate to public WTA compensation, mainly for lost ES due to hydropower dam building in the Brazilian Amazon [44, 59, 61]. These average WTA values (1,461 USD/ha/year) are significantly higher than the 22 stated WTP values (362 USD/ha/year) for the protection of ES based on the Mann-Whitney test (MW Z = 2.446; *p*<0.013). This confirms previous findings in the stated preferences literature examining WTP-WTA discrepancies [e.g. 73]. Although much higher, the CV-based WTA values (1461 USD/ha/year) are not significantly different from the CE-based WTA values (56 USD/ha/year) extracted from [39] for the loss of the hydrological services provided by the Amazon river due to an oil spill at the 10 percent significance level (MW Z = -1.709; *p*<0.117). This is mainly due to the low number of observations and the wide range of CV-based WTA values (1.0–4,355 USD/ha/year).

The mean value for the two PES observations, where forest dwellers are paid for the protection of habitat for species in the reserve Chico Mendes in Xapuri (Acre) and carbon credits in the city of Presidente Figueiredo (Amazonas), is 70 percent lower than public WTP for these ES (362 USD/ha/year). However, due to the low number of observations for PES, this difference is not statistically significant (MW Z = -0.731; *p*<0.522). PES was included as a separate valuation method because it is not exactly the same as market prices. The payment levels are often politically negotiated and represent financial compensations to landowners to cover their opportunity cost of participation in a PES scheme and an incentive payment [e.g. 74]. Both studies focusing on PES [54, 66] examined what should be paid for the ES involved to incentivize and sustain participation. The average PES value is around 10 percent lower than the value based on market prices and their difference is not statistically significant (MW Z = 0.794; *p*<0.500).

Public WTP to preserve the ES using the CV method is 30 percent higher than the avoided damage costs, but also not significantly different (MW Z = 1.589; *p*<0.128). Similarly, also the difference between PES and avoided damage costs is not significant (MW Z = 1.155; *p*<0.400) even though the avoided damage costs related to the protection of ES are four times higher than the two PES values. Note that most of the avoided damage cost estimates relate to the beneficial effects of carbon sequestration on moderating the negative impacts of climate change (droughts, heat waves, flooding) and property damage. The latter can be very high.

The value estimates presented in Table 1 were also analyzed for the whole and parts of the Brazilian Amazon in different states. The mean value estimate is 270 USD/ha/year (with a standard error of 160 based on 6 observations) in case the whole Brazilian Amazon is valued and 429 USD/ha/year for parts thereof across different states (with a standard error of 137 based on 47 observations). The difference is, however, not statistically significant (MW Z = 0.730; *p*<0.484). The opposite result is found in an international CV study [20], in which the whole Brazilian Amazon was valued significantly higher than its parts. Their aggregate CV-based value in the UK and Italy for the preservation of 5 percent of the Brazilian Amazon was 68 USD/ha/year in 2020 prices. Only two CV studies value the Brazilian Amazon forest as a whole [31, 46]. The average of these two CV values for the Amazon as a whole is 51 USD/ha/year, whereas mean CV-based WTP for the preservation of parts of the Brazilian Amazon as habitat for species across Acre, Amazonas, Maranhão, Mato Grosso, Pará, Roraima and Tocantins is 318 USD/ha/year based on 14 observations from 12 studies. This latter value is almost five times higher than the aggregate value based on [20]. Finding higher local than national values for the ES involved may be due to possible place attachment [e.g. 75, 76].

Comparing these values with other stated preference values found for the Amazon in the existing international literature, especially in Europe, confirms that these aggregated international values are much lower. Based on our own calculations, preservation of 20 percent of the whole Amazon in a CV survey among households in Madrid, Spain [21] results in an aggregate value over all households in Madrid of 0.6 USD/ha/year in 2020 prices. The aggregate non-use value over all Norwegian households reported in [22] is similarly low (0.2 USD/ha/year in 2020 prices) for the preservation of 85 percent of the whole Amazon in South America, while the estimate presented in [23] amounts to an aggregate value of 0.6 USD/ha/year in 2020 prices based on the application of the Delphi method, asking valuation experts around the world for the non-use values of the entire Amazon rainforest in South America. These relatively low values can possibly be explained by the fact that the local values consist of use and non-use values held by both users and nonusers, while at the same time place attachment is, as said, expected to play a prominent role in our study too.

### 4.3. Meta-regression model results

Table 2 presents the results when regressing the economic values detailed in the previous section on multiple explanatory factors in a GLS regression model that accounts for heteroscedasticity due to the fact that several studies generated multiple observations. Four different models are presented. The first three models include the values for all ES, while the fourth model zooms in on the stated preference valuation results for the ES ‘habitat for species’. The numbers of observations for the other ES are too small to conduct a meaningful analysis. The first two models (models I and II) are based on all values extracted from the Brazilian valuation literature (n = 61), whereas model III excludes the outliers to test the robustness of the results. This model is directly based on the economic values summarized in the previous section (n = 53). Although it is common practice to combine ES and valuation methods in meta-regression models [e.g. 77–81], there may exist significant correlation between some of the ES and the methods used to value them. We therefore initially omitted the explanatory variables for the valuation methods in the first meta-regression model, and include them in the second and third model, together with the type of welfare measure (WTP or WTA). Correlation is only detected between the travel cost method and recreation (r = 0.470) and avoided damage costs and carbon sequestration (r = 0.428). However, the number of observations for each of these combinations is low (only 2 observations in each case), and therefore not expected to play a very significant role. In model IV, we include a dummy variable for the type of stated preference method (CV or CE) and welfare measure (WTP or WTA).

**Table 2:** Generalized least squares regression results.

		Model I	Model II	Model III	Model IV
		*All ES*, *all observations*^a^	*All ES*, *all observations*^a^	*All ES*, *excl*. *outliers*^a^	*Habitat only*, *excl*. *outliers*^b^
**Dependent variable:**	**Mean**		**Coeff. est.**	**Coeff. est.**	**Coeff. est.**
Natural log 2020 USD/ha/year	**(St. dev.)**		**(St. error)**	**(St. error)**	**(St. error)**
Constant	--	5.121***	0.693	-1.704**	-3.659**
		(1.544)	(2.102)	(0.837)	(1.587)
**Study variables**					
Study year	11.049	0.259***	0.401***	0.243***	-0.127*
	(6.273)	(0.042)	(0.082)	(0.046)	(0.067)
Peer reviewed paper (D)	0.541	-1.177**	0.815	-0.881**	0.362
	(0.502)	(0.580)	(0.798)	(0.389)	(0.361)
**Valuation methods** (baseline category models II and III: stated preferences; model IV: contingent valuation)					
Choice experiment (D)	0.049	--	--	--	2.800***
	(0.218)				(1.015)
WTA (D)	0.164	--	2.129***	2.953***	1.771**
	(0.373)		(0.712)	(0.255)	(0.730)
Travel costs (D)	0.066	--	-0.116	-0.356	--
	(0.250)		(0.902)	(1.467)	
Payments for ES	0.033	--	4.659***	3.708***	--
	(0.180)		(1.914)	(1.294)	
Market prices (D)	0.246	--	-2.200***	-0.541	--
	(0.434)		(0.657)	(0.641)	
Avoided damage costs (D)	0.049	--	-0.821	0.691	--
	(0.218)		(1.384)	(1.364)	
**Ecosystem services** (baseline category models I-III: habitat for species)					
Carbon regulation (D)	0.098	0.377	1.715*	1.769***	--
	(0.300)	(0.522)	(0.937)	(0.529)	
Water cycling (D)	0.066	-0.909*	0.469	0.464	--
	(0.250)	(0.520)	(0.962)	(0.522)	
Recreation and ecotourism (D)	0.213	0.831	4.826***	1.289*	--
	(0.413)	(0.837)	(0.818)	(0.714)	
**Drivers of change (threats)** (baseline category: general environmental degradation)					
Deforestation (D)	0.197	2.314***	0.264	-0.667	-0.838
	(0.401)	(0.629)	(0.969)	(0.790)	(0.645)
Mining (D)	0.049	0.458	-1.494	-2.895***	-1.925***
	(0.218)	(0.728)	(1.138)	(0.692)	(0.680)
Wildfires (D)	0.033	3.326***	5.140***	2.736***	--
	(0.180)	(0.773)	(1.295)	(0.549)	
Hydro dams (D)	0.082	4.869***	4.899***	4.590***	-1.881*
	(0.277)	(0.878)	(1.392)	(0.520)	(1.075)
River pollution (D)	0.164	3.698***	4.292***	2.319***	-2.766***
	(0.373)	(0.577)	(1.287)	(0.462)	(1.008)
**Location details**					
Area size (1,000 ha’s) (nat. log)	57,966	-0.691***	-0.762***	-0.357***	-0.089
	(132,270)	(0.055)	(0.044)	(0.032)	(0.075)
Whole Brazilian Amazon (D)	0.082	2.661***	5.944***	2.827**	--
	(0.277)	(0.959)	(1.513)	(1.447)	
Protected area (D)	0.443	0.002	0.786	1.446**	-1.922***
	(0.501)	(0.597)	(0.676)	(0.623)	(0.610)
Beach (D)	0.164	-1.216	-3.566***	-3.646***	--
	(0.373)	(0.851)	(0.672)	(0.484)	
**Population of beneficiaries**					
Population density (inhabitants/km^2^) (nat. log)	6.515	1.531***	1.720***	2.525***	3.525***
	(19.836)	(0.260)	(0.478)	(0.314)	(0.695)
Monthly income (D)	0.885	1.155**	2.943***	1.735***	5.333***
(1 if larger than USD 250/capita)	(0.321)	(0.515)	(0.754)	(0.263)	(1.139)
**Summary statistics**					
Number of clusters (study level)		36	36	32	19
Wald chi-squared test statistic		7,369.23***	2,159.50***	18,851.79***	3,757.06***
Pseudo R^2^		0.407	0.463	0.379	0.383
Relative MAPE (%)^c^		56.0	50.7	48.2	56.1
Number of observations		61	61	53	26

Notes

^a^ All ES: all ecosystem services covered in the studies included in the meta-analysis.

^b^ Habitat for species only.

^c^ Mean absolute prediction error.

All models include main study characteristics, such as the valued ES, the identified threats to which the ES are exposed in the valuation study, whether the study valued a part or the whole of the Brazilian Amazon, the year in which the study was conducted to account for possible time trends and whether or not the study was published in a peer reviewed journal. External explanatory factors typically found in similar meta-analyses of environmental valuation studies [e.g. 1, 4, 82, 83], such as the scale of analysis (the amount of forest under valuation), whether the study area is located in a protected conservation area, the type of biome (whether the study focused on natural beaches or other) and the size and characteristics (income) of the populations benefiting from the ES are also included in all models.

The last model, capturing stated preference values for ‘habitat for species’, includes the same set of covariates as the three other models except the dummy variables for the ES and valuation methods. The valuation scenarios applied in these 19 stated preference studies were very generic. As a consequence, no additional information could be extracted from these studies about habitat type, species or species abundance, as for example in a similar meta-analysis focusing on different ecosystems in [84]. The dummy variables for natural beaches and wildfires were excluded in this last model because they refer more to use related values, including public health, while the dummy for the whole Amazon dropped out due to multicollinearity.

Comparing models I and II, including control for the valuation methods increases both the explanatory and predictive power of model II. PES generates significantly higher values than the baseline category which consists of stated preference values, whereas market prices yield significantly lower ES values. Values based on travel costs and avoided damage costs also produce lower values, but they do not differ significantly from the stated preference values. The latter result takes away some of the concerns surrounding their correlation with ES. Carbon sequestration and ecotourism exhibit significantly higher values than habitat for species (the baseline category), whereas water regulation is not significantly different. Water regulation is the only ES that is statistically significant in model I at the 10 percent level. Otherwise, the two models are fairly similar. Different is that the constant term, the dummy for peer reviewed journal articles and deforestation are significant in model I, but not in model II, while the beach dummy is significant in model II and not in model I.

The results for models II and III are even more consistent. Model III confirms that there exists a significant time trend. The positive coefficient indicates that the longer ago a study was carried out, the higher the estimated economic value. Hence, whilst controlling for other influencing factors, the estimated values for the ES have become smaller over time, with more recent studies generating, on average, lower values. One possible explanation for this is that the provision and quality of the ES, and hence their values, have eroded over time. Another explanation is that the applied research methodologies have advanced over time and increasingly generated more conservative estimates. Jumping ahead to model IV, the average value attached to habitat for species has increased over time, signaling an increase of scarcity in the provision level of this specific ES. A negative publication bias can be observed at the same time in model III (as in model I), indicating that valuation results that have been published in peer reviewed journals are, on average, significantly lower than results extracted from reports or other non-peer reviewed publications. No significant publication bias can be detected in model IV for stated preference research on habitat preservation in the Brazilian Amazon. In model IV control is included for the stated preference valuation approach. CE yields significantly higher values than CV whilst controlling for the different types of welfare measures. As expected, asking Brazilian respondents for their WTA compensation for the lost ES instead of their WTP to conserve them (the baseline category) results on average and all else equal in significantly higher economic values due among others to loss aversion [84].

Carbon regulation and recreation and ecotourism are valued significantly higher than habitat for species once control is included for the valuation methods, irrespective of the inclusion or exclusion of outliers. However, the difference between the coefficient estimates in model III is statistically not significant (Wald chi-square test statistic is 0.45; *p*<0.501), meaning that their values are the same. Actual payments for ES are also in model III significantly higher than stated preferences for ES. Market prices maintain a negative sign in model III, but the coefficient is not statistically significant.

Consistent across all three models (1-III) are the impacts of most drivers of change, especially wildfires, the construction of hydro-electric dams and water pollution. The same applies to the size of the area providing the ES, whether part or the whole Brazilian Amazon is valued, population density and per capita income. Dam building is considered the most important threat in most models, followed by wildfires and river pollution. The coefficient estimates of the latter two are not significantly different in model III (Wald chi-square test statistic is 0.69; *p*<0.406). Compared to general environmental degradation (the baseline category), only mining is considered significantly less important, also in the last model IV, where now also hydropower dams and water pollution are considered less important than general environmental degradation. Deforestation only has a significant positive effect in model I.

The few studies that valued the whole Brazilian Amazon instead of its parts produce, on average and all else equal, significantly higher values in models I-III. In studies where parts of the Amazon are valued, smaller areas are valued more than larger areas across all four models. The economic value hence decreases as the size of the area is increased, possibly due to place attachment where significantly higher values are attributed to the preservation of ES that are enjoyed locally. The emphasis on local values can be observed more generally in much if not most of the reviewed literature and is perhaps also somewhat inherent to the focus of the meta-analysis. The dummy for protected areas has a positive sign in the first three models, but is only statistically significant in model III. A sign switch is furthermore observed in model IV, indicating that stated preferences for the preservation of species habitat increase if the study area does not (yet) have an official protected status.

Population density in the areas where the population of beneficiaries live significantly increases the economic value in all four meta-regression models. The estimated coefficient is, as expected, positive: the higher the population density and hence demand, the higher the economic value for the ES provided by the Brazilian Amazon rainforest. A one percent increase in population density results all else constant in an increase in the average unit value between 0.9 percent for all ES and 1.3 percent for habitat for species. Equally significant and positive is the impact of the income level of the population living in the municipalities where the ES are provided. The coefficient estimates show that if average disposable household income in a municipality benefitting from the ES is higher than the median income value for all municipalities in the database, which is more or less equal to the average minimum wage in Brazil over the period 1995–2020, this results, on average and all else equal, in a 0.6 percent increase in the average economic value for all ES and 1.7 percent for habitat for species.

Finally, the predictive power of the estimated models was also estimated based on the leave-one-out resampling procedure. The predictive power is measured by the relative mean absolute prediction error (MAPE). As can be seen in the lower part of Table 2, this relative error is lowest for model III. These errors indicate how much predictions based on the estimated models would over- or underestimate the actually observed values in the different case studies, on average. The values for the regulating and cultural ES predicted based on model III will be 48 percent higher or lower than the actually observed values, whereas this is 56 percent for species habitat. Although such errors are not uncommon in the literature [e.g. 82, 85], they question the reliability of employing the estimated meta-regression models for the purpose of value transfer and upscaling across areas where no original valuation research has been conducted and calculate the total economic value of the Brazilian Amazon for the ES involved. It should be noted that the prediction errors of the estimated meta-regression models are lower than those for simple mean value transfer. The latter errors are based on the use of sample averages without control for influencing factors to predict values and this generates errors that are 25 to 45 percent higher than if the estimated meta-regression models are used. These results are summarized in S5 Table in S1 File.

## 5. Discussion and conclusions

Although the largest rainforest in the world and home to the highest diversity in flora and fauna, a systematic review and analysis of the economic valuation literature related to the Amazon is strikingly missing. This study fills this gap by screening and analyzing the existing Brazilian valuation literature, predominantly in Portuguese and not published internationally, focusing on an important sub-set of domestic market and non-market values of regulating and cultural ES provided by the Brazilian Amazon rainforest, benefitting local populations and communities.

The costs of protecting the Amazon consist mainly of the agricultural income lost to the Brazilian economy. These costs have been estimated in various studies in the past [e.g. 86], most recently perhaps by [87] at 797 USD/ha/year for the Legal Brazilian Amazon in 2006. In 2020 price levels, this latter estimate of the opportunity costs of preserving the Brazilian Amazon would be four times higher than the estimated mean value found in this study (USD 411/ha/year) for the regulating and cultural ES provided by the Amazon rainforest to local populations and communities. Although the estimated economic values vary considerably across different ES and do not exceed this opportunity cost estimate, especially the substantial values for recreation and ecotourism (410 USD/ha/year) and habitat for species (455 USD/ha/year) show that Brazilians are willing to pay a substantial amount of money for the use and nonuse values associated with the preservation of the Amazon. These local values are higher than the values elicited outside Brazil, for example in Europe for the preservation of the Brazilian Amazon, and should be accounted for in cost-benefit assessments underpinning policy and decision-making related to the preservation of the Brazilian Amazon forest. They should furthermore be added, for example, to the present values estimated for the Brazilian Amazon’s provisioning services in [15], which varied between 57 and 737 USD/ha/year.

An important question remains whether the estimated values for the different ES in this study can be aggregated to obtain a total economic value for the Brazilian Amazon rainforest. The sum of the mean values presented in Table 1 for the different ES comes close to the opportunity cost of Amazon preservation. However, one of the challenges in this study was the identification and disentanglement of the various ES valued in the Brazilian literature. Many of the ES, if not most, are supplied jointly, not separately, and treating them independently can therefore be an analytical artefact. In some studies, they were valued individually, but in a number of cases arbitrary choices had to be made to assign values to ES separately. The definition of the specific ES in the existing valuation literature and also their relationship to biodiversity remains an important conceptual and empirical issue [e.g. 6, 88, 89]. Treating ES separately increases the risk of double counting when adding them up. Addressing the question how their joint delivery can be valued in conjunction with their impact on and overlap with biodiversity, using geographical information systems, would be an important step forwards to inform and spatially target conservation efforts in the Brazilian Amazon.

Another important question is how reliable the synthesized values presented here are for value transfer and upscaling purposes. The broad range of values extracted from the different valuation studies, their relatively high measurement errors and the limited prediction power of the estimated meta-regression models (±50%) somewhat reflect the lack of a coherent, common valuation and ES accounting framework for the Brazilian Amazon rainforest. Each study in our database used its own valuation approach with limited cross-referencing to existing studies. The quality of the applied valuation studies varies, especially among those applying non-market valuation methods. These non-market valuation studies are responsible for two thirds of all values in our meta-analysis. Many of them provide limited information about what is often deemed essential to evaluate the validity and reliability of the valuation results, and not all seem to follow state-of-the-art guidelines for non-market valuation [e.g. 90]. For example, only a limited number of the studies reported whether their valuation design, including the selected bid values in the dichotomous choice CV studies, had been pretested, response and protest rates were reported sporadically, while several studies had very low sample sizes of 40–60 respondents only. Although all stated preference studies were conducted after 1995, only one study used a CE, the most commonly used stated preference method since the mid-1990’s [30]. And only about half of the CV studies used the dichotomous choice elicitation format recommended by the NOAA Panel [91], while for approximately 10 percent of these CV studies the applied elicitation format could not be identified because it was not reported and could also not be inferred from the data analysis.

In conclusion, there exists a need for more peer reviewed valuation research in large parts of the Brazilian Amazon where values are currently lacking to enable reliable extrapolation and upscaling of the economic values into a total economic value for the Amazon as a whole. The considerable prediction errors of the estimated meta-regression models undermine their practical applicability to inform forest conservation policy and decision-making. Future valuation research should be guided by an agreed upon common valuation framework for the Amazon, using state-of-the-art valuation guidelines, based on a standardized ES accounting system to make future values more comparable, reduce study and data heterogeneity, and increase the explanatory and predictive power of future meta-regression models for the purpose of value transfer and spatial upscaling. Existing global meta-analyses of economic valuation studies of forest ES provide only limited guidance due to variations in the categorization of ES and the use of different biodiversity indicators. A new accounting and valuation framework for the Amazon therefore seems needed.

## Supporting information

S1 Checklist (DOCX)

S1 File (DOCX)
