# Supplementary material for: The economic value of the Brazilian Amazon rainforest ecosystem services: A meta-analysis of the Brazilian literature
Source: PLoS One. 2022 May 19;17(5):e0268425. doi: 10.1371/journal.pone.0268425 (PMC9119521; doi:10.1371/journal.pone.0268425)
Supplement: S1 File — (DOCX) [file pone.0268425.s002.docx]

**Supporting Information**

**Table S1. Studies included in the meta-analysis in alphabetical order of authorship**

| **Study nr.** | **Bibliographic details** |
| --- | --- |
| 1 | Alves, C.S., Grzebieluckas, C., 2013. Estimativa do valor econômico dos serviços ambientais pelo método custo de viagem: um estudo na Cachoeira Salto das Nuvens em Tangará da Serra MT. XIII Congresso de Custos 18-19 Abril 2013, Porto, Portugal. |
| 2 | Alves, G.L., Paixao, A.N., 2012. Efeitos das queimadas sobre o bem-estar das famílias no Tocantins: uma aplicação do método de avaliação contingente. Informe Gepec 16(1), 176-191. |
| 3 | Amend, M.R., Reid, J., Gascon, C., 2006. Benefícios econômicos locais de áreas protegidas na região de Manaus, Amazonas. Megadiversidade 2(1-2), 60-70. |
| 4 | Bentes, E.S., Santana, A.C., Homma, A.K.O., Gomes, S.C., 2014. Valoração Econômica da jusante da barragem de Tucuruí. Revista de Política Agrícola XXIII 4, 102-110. |
| 5 | Bocato Junior, F.C., 2009. Valoração econômica de activos naturais urbanos: o caso da área de preservação ambiental da Fazendinha e seu entorno, Macapá - Amapá. Tese de Mestrado. Universidade Federal do Amapá. 101pp. |
| 6 | Fearnside, P.M., 2000. O potencial do setor florestal Brasileiro para a mitigação do efeito estufa sob o 'Mecanismo de Desenvolvimento Limpo' do Protocolo de Kyoto. pp.59-74. *In*: Moreira, A.G., Schwartzman, S. (eds.) As Mudanças Climáticas e os Ecossistemas Brasileiros. Instituto de Pesquisa Ambiental da Amazônia (IPAM), Woods Hole Research Center and Environmental Defense, Brasilia, DF. 165pp. |
| 7 | Fearnside, P.M., 2003. Desenvolvimento Sustentável e serviços ambientais na Amazônia. XXVII ANPOCS - GT 11- O Desenvolvimento Sustentável em Questão na Amazônia Brasileira, Caxambu-MG, 21-26/10/03. |
| 8 | Finco, M.V.A., Rodrigues, W., Rodrigues, I., 2006. A Amazônia Legal Brasileira e o mercado de créditos de Carbono: perspectivas para o estado do Tocantins. Amazônia: CI & Desenv. 2(3), 7-24. |
| 9 | Finco, M.V.A., Valadares, M.B., 2008. Estimando o valor de uso do meio ambiente na Amazônia Legal: uma aplicação do método do custo de viagem às Praias de Palmas, Tocantins. Amazônia: Ci. & Desenv. 3(6), 147-161. |
| 10 | Fonseca Filho, L.A.F., 2012. Modelo de gestão econômico-ambiental por renumeração de serviços ambientais por créditos de carbono, no município de Presidente Figueiredo/AM, com simulação de SAFS. Master Thesis, Universidade Federal do Amazonas, Manaus, Amazonas, 69p. |
| 11 | Freitas, K.A.A., Filho, J.B., Pio, N.S., Silva, F.F., Moraes, L.S., 2010. Valoração econômica dos benefícios ambientais percebidos pela população da bacia do Educandos provenientes do PROSAMIM. Acta Amazônica 40(3), 509-514. |
| 12 | Freitas, E.A.S.F., Maia, J.C.S., Pereira, B.D., 2003. Valoração econômica de ativos ambientais na suinocultura: um estudo de caso no município de Diamantino-MT. Congresso SOBER 2003. |
| 13 | Gutierrez, C.B.B., Gutierrez, D.M.G., Santos, L.S., Dias, N.M., Ribeiro, H.M.C., Morales, G.P., 2016. Disposição a Pagar pelo Uso e Conservação de um Lago no Cenário Amazônico: O Caso do Lago da Coca-Cola, Salinópolis - Pará. Revista Espacios 37(19), 28. |
| 14 | Hage, A.F.A., Beltrao, N.E.S., Tavares, P.A., Pena, H.W.A., 2017. Disposição a pagar por recursos naturais na Amazônia: Imediações do porto de Camará, Salvaterra /Monsarás -PA. Revista Contribuciones a las Ciencias Sociales ISSN: 1988-7833 (http://www.eumed.net/rev/cccss/2017/04/recursos-naturais-amazonia.html) |
| 15 | Maciel, R.C.G., Reydon, B.P., Costa, J.A., Sales, G.O.O., 2010. Pagando pelos Serviços Ambientais: Uma proposta para a Reserva Extrativista Chico Mendes. Acta Amazônica 40(3), 489-498. |
| 16 | Magalhães Filho, L.N.L., Vergara, F.E., Rodrigues, W., 2012. Valoração de danos ambientais devido à implantação de UHE de Estreito: o caso de Babaçulândia - Tocantins. Espaço Energia 16, 1-10. |
| 17 | Mendonça, A.F., Tilton, J.E., 2000. A Contingent Valuation Study of the Environmental Costs of Mining in the Brazilian Amazon. Minerals & Energy 15, 21-32. |
| 18 | Nascimento, A.R.C., Grzebieluckas, C., Santos, J.S.C., 2018. Pagamento por Serviços Ambientais – PSA: Um estudo sobre a Sub-Bacia do Rio Queima Pé em Tangará da Serra-MT. 8° Congresso UFSC de Controladoria e Finanças, Florianópolis, SC, Brasil, 12 - 14 August 2018. 16p. |
| 19 | Nifossi, V.S., Grzebieluckas , C., Santos, J.S.C., Socoloski, A., 2018. Valoração Contingente: quanto a população está disposta a pagar pela recuperação e manutenção de áreas verdes? 8° Congresso UFSC de Controladoria e Finanças, Florianópolis, SC, Brasil, 12 - 14 August 2018. 15p. |
| 20 | Nishimura, C.N., Campos, S.P., Tavares, P.A., Betrao, N.E.S., 2017. Aplicação do Método de Avaliação Contingente de uma Unidade de Conservação: Estudo de Caso do Museu Parque Seringal-PA. XII Encontro Nacional da Sociedade Brasileira de Economia Ecológica, 19-22 September, 2017. Universidade Federal de Uberlandia (MG), 13p. |
| 21 | Oliveira, F.T., 2014. Desafios do serviço florestal de ecoturismo no Brasil: perspectivas de desenvolvimento nas florestas nacionais da Amazônia. Tese de Doutoramento Brasilia/DF. PGEFL.TD-043/2014. 143pp. |
| 22 | Pereira, A.M.A., 2008. Valoração econômica dos impactos ambientais em assentamentos rurais de Rorainópolis - RR. Dissertação de Mestrado. Universidade Federal do Amazonas, Programa de Pós-graduação em Ciências Florestais e Ambientais. Manaus - AM, 159pp. |
| 23 | Pessoa, R., Ramos, F.S., 1998. Avaliação de Ativos Ambientais: Aplicação do Método de Avaliação Contingente. RBE Rio de Janeiro 52(3), 405-426. |
| 24 | Ribeiro, M.R.R., 2011. Valoração econômica dos ativos ambientais provenientes de uma floresta tropical. Master thesis. Manaus: UFAM, 2011, 64pp. |
| 25 | Rivas, A., Casey, J.F., Kahn, J.R., 2006. A preservação ambiental é um bem de luxo? Um estudo sobre valor de ecossistema na Amazônia. Planejamento e Políticas Públicas 29, 39-55. |
| 26 | Rodrigues, W., Souza, E.C., 2008. Valoração dos danos ambientais causados pela implantação da usina hidroeléctrica Luis Eduardo Magalhães no municipio de Porto Nacional: uma aplicação do método de valoração contingente. SOBER XLVI Congresso da Sociedade Brasileira de Economia, Administração e Sociologia Rural. Rio Branco – Acre, 20 a 23 de Julho de 2008, 15p. |
| 27 | Rodrigues, W., Magalhães Filho, L.N., Figueroa, F.V., 2013. Valoração dos danos ambientais advindos da construção de hidroelectricas: o caso da UHE de Estreito. Informe Gepec 17(2), 23-39. |
| 28 | Santana, R.F., Mota, J.A., 2004. Economia e valor de existência: O caso do Parque Nacional do Jaú (Amazonas). Texto para discussão nº1008. Brasília ISSN 1415-4765. |
| 29 | Santana, A.C., Salomão, R.P., Santana, A.L., Castilho, A.F., 2016. O valor econômico total da area de savana metalófita, ou “canga”, da floresta nacional de Carajás, estado do Para: uma contribuição teórica e metodológica da avaliação contingente. Papers do NAEA nº 361, ISSN 15169111, 48pp. |
| 30 | Santana, A.C., Santana, A.L., Santana, A.L., Gomes, S.C., Salomão, R.P., 2015. Valoração dos danos ambientais causados por hidrelétricas para a produção de energia na bacia do Tapajós. Reflexões Econômicas 1(1), 31-48. |
| 31 | Seroa da Motta, R., 2002. Estimativa do Custo Econômico do Desmatamento na Amazônia. Texto para Discussão n°910, ISSN 1415-4765, 29pp. |
| 32 | Silva, R.G., 2018. Avaliação econômica dos serviços ambientais da Bacia do Rio Acre. Inclusão Social 12(1), 152-160. |
| 33 | Silva, R.G., Lima, J.E., 2004. Valoração Contingente do Parque 'Chico Mendes': uma Aplicação Probabilística do Método Referendum com Bidding Games. RER 42(4), 685-709. |
| 34 | Silva, R.G., Lima, J.E., 2006. Avaliação econômica da poluição do ar na Amazônia Ocidental: um estudo de caso do Estado do Acre. RER, 44(2), 157-178. |
| 35 | Sousa, T.B., Cunha, E.B., 2013. Valoração econômica ambiental: uma estimativa do valor de uso e valor de nao uso do Rio Amazonas no litoral da capital amapaense. Revista Meio Ambiente e Sustentabilidade 4(2), 265-286. |
| 36 | Zacarkim, C.E., 2012. Diagnóstico da pesca artesanal e amadora no Rio Araguaia - TO/PA. Tese de Doutoramento, Curitiba 2012, CDU 639.2, 61pp. |

**Table S2. Overview of study characteristics**^1^

| **Study** | **Publication type**^2^ | **Language**^3^ | **Published in English?** | **Study year(s)** | **Location (state or whole Amazon)** | **Valuation method**^4^ | **Ecosystem services**^5^ | ***N***^6^ |
| --- | --- | --- | --- | --- | --- | --- | --- | --- |
| 1 | NPR paper | P | No | 2012 | Mato Grosso | TC | CES | 1 |
| 2 | PR paper | P | No | 2009-2010 | Tocantins | CV | Habitat | 1 |
| 3 | PR paper | P | No | 2003 | Amazonas | MP | Habitat | 10 |
| 4 | PR paper | P | No | 2011 | Pará | CV | Habitat | 1 |
| 5 | Thesis | P | No | 2008-2009 | Amapá | CV | CES | 1 |
| 6 | Book chapter | P | No | 1997 | Whole Amazon | ADC | CR | 1 |
| 7 | NPR paper | P | Yes | 1990 | Whole Amazon | ADC | CR, HC | 2 |
| 8 | PR paper | P | No | 2004 | Tocantins | MP | CR | 2 |
| 9 | NPR paper | P | No | 2005 | Tocantins | CV and TC | CES | 4 |
| 10 | Thesis | P | No | 2012 | Amazonas | PES | CR | 1 |
| 11 | PR paper | P | No | 2008 | Amazonas | CV | Habitat | 1 |
| 12 | NPR paper | P | No | 2002 | Mato Grosso | CV | Habitat | 1 |
| 13 | PR paper | P | No | 2015 | Pará | CV | CES | 1 |
| 14 | PR paper | P | No | 2017 | Pará | CV | Habitat | 1 |
| 15 | PR paper | P | No | 2010 | Acre | PES | Habitat | 1 |
| 16 | PR paper | P | No | 2008 | Tocantins | CV-WTA | Habitat | 1 |
| 17 | PR paper | E | Yes | 1997 | Pará | CV | Habitat | 3 |
| 18 | NPR paper | P | No | 2017 | Mato Grosso | CV-WTP&WTA | HC | 2 |
| 19 | NPR paper | P | No | 2017 | Mato Grosso | CV | Habitat | 1 |
| 20 | NPR paper | P | No | 2015 | Pará | CV | CES | 1 |
| 21 | Thesis | P | No | 2012 | Pará | CV and TC | CES | 2 |
| 22 | Thesis | P | No | 2007 | Roraima | CV | Habitat | 2 |
| 23 | PR paper | P | No | 1995 | Roraima | CV | Habitat | 1 |
| 24 | Thesis | P | No | 2010 | Amazonas | CV-WTA | Habitat | 1 |
| 25 | PR paper | P | No | 2003 | Amazonas | CE-WTA | Habitat | 3 |
| 26 | NPR paper | P | No | 2006 | Tocantins | CV-WTA | Habitat | 1 |
| 27 | PR paper | P | No | 2008 | Maranhão and Tocantins | CV-WTA | Habitat | 1 |
| 28 | Report | P | No | 2003 | Amazonas and Roraima | CV | Habitat | 1 |
| 29 | NPR paper | P | No | 2012 | Pará | CV-WTP&WTA | Habitat | 2 |
| 30 | PR paper | P | No | 2014 | Pará | CV-WTP&WTA | Habitat | 2 |
| 31 | Report | P | Yes | 2000 | Mato Grosso & whole Amazon | CV, MP | Habitat, CR, CES | 3 |
| 32 | PR paper | P | No | 2009 | Acre | CV | HC | 1 |
| 33 | PR paper | P | No | 2002 | Acre | CV | Habitat | 1 |
| 34 | PR paper | P | No | 2004 | Acre | CV | Habitat | 1 |
| 35 | PR paper | P | No | 2012 | Amapá | CV | CES | 1 |
| 36 | Thesis | P | No | 2009 | Pará and Tocantins | MP | CES | 1 |

^1^ Study numbers correspond to the numbering of studies in Table S1 in the Supporting Information. ^2^ NPR: non-peer reviewed; PR: peer reviewed. ^3^ P: Portuguese; E: English. ^4^ CV: contingent valuation; CE: choice experiment; WTA: willingness to accept compensation; TC: travel costs; MP: market prices; PES: payments for ecosystem services; ADC: avoided damage costs. ^5^ Habitat: habitat for species; CR: carbon regulation; HC: hydrological cycle; CES: cultural ecosystem services (eco-tourism and recreation). ^6^ Number of observations extracted from each study.

.

**Table S3. Economic values extracted from the studies included in the meta-analysis, their original measurement units and associated population and area size**^1^

| **Study no.** | **Obs.** | **Original value** | **Measurement unit** | **Study year** | **Population size** | **Area size (ha)** | **Comment** |
| --- | --- | --- | --- | --- | --- | --- | --- |
| 1 | 1 | 1,049,400 | R$/year | 2012 | Aggregated value across all visitors (number not specified) | 716,804 | Not reported in the study |
| 2 | 1 | 21,924,648 | R$/year | 2009 | Aggregated over 280,223 households | 28,000,000 | The state of Tocantin |
| 3 | 1 | 141.11 | USD/ha/yr | 2003 | Population (number not specified) living around the region of Manaus | 2,488 | Reported in the study |
| 3 | 2 | 0.16 | USD/ha/yr | 2003 | Population (number not specified) living around the region of Manaus | 350,018 | Reported in the study |
| 3 | 3 | 352.1 | USD/ha/yr | 2003 | Population (number not specified) living around the region of Manaus | 500 | Reported in the study |
| 3 | 4 | 0.18 | USD/ha/yr | 2003 | Population (number not specified) living around the region of Manaus | 2,272,000 | Reported in the study |
| 3 | 5 | 0.02 | USD/ha/yr | 2003 | Population (number not specified) living around the region of Manaus | 157,807 | Reported in the study |
| 3 | 6 | 12,970.1 | USD/ha/yr | 2003 | Population (number not specified) living around the region of Manaus | 33 | Reported in the study |
| 3 | 7 | 4.31 | USD/ha/yr | 2003 | Population (number not specified) living around the region of Manaus | 760 | Reported in the study |
| 3 | 8 | 3.12 | USD/ha/yr | 2003 | Population (number not specified) living around the region of Manaus | 18,900 | Reported in the study |
| 3 | 9 | 15.95 | USD/ha/yr | 2003 | Population (number not specified) living around the region of Manaus | 9,572 | Reported in the study |
| 3 | 10 | 0.14 | USD/ha/yr | 2003 | Population (number not specified) living around the region of Manaus | 943,000 | Reported in the study |
| 4 | 1 | 55,440,940.85 | R$/year | 2011 | Aggregated across all fishermen in the area (170,093) | 770,980 | Not reported in the study |
| 5 | 1 | 634,752 | R$/year | 2008 | 57,600 visitors per year | 193 | Reported in the study |
| 6 | 1 | 4,000 | USD/ha | 1990 | Aggregated over hectares (population not specified) | 420,000,000 | The whole Brazilian Amazon |
| 7 | 2 | 70.7 | USD/ha/yr | 1990 | Aggregated over hectares (population not specified) | 420,000,000 | The whole Brazilian Amazon |
| 7 | 5 | 19 | USD/ha/yr | 1990 | Aggregated over hectares (population not specified) | 420,000,000 | The whole Brazilian Amazon |
| 8 | 1 | 1,265,720 | USD/yr | 2004 | Aggregated gains to the state Tocantins due to conservation units (population not specified) | 15,821.5 | Reported in the study |
| 8 | 3 | 90802.4 | USD/yr | 2004 | Aggregated gains to the state Tocantins due to conservation units (population not specified) | 1,135.0 | Reported in the study |
| 9 | 1 | 256,200 | R$/month | 2005 | 120 visitors | 4.7 | Not reported in the study |
| 9 | 2 | 108,784.17 | R$/month | 2005 | 120 visitors | 4.7 | Not reported in the study |
| 9 | 3 | 270,240.03 | R$/4 monts | 2005 | 120 visitors | 4.7 | Not reported in the study |
| 9 | 4 | 94,463.43 | R$/4 monts | 2005 | 120 visitors | 4.7 | Not reported in the study |
| 10 | 1 | 2,496.35 | R$/ha | 2012 | Local farmers in municipality Presidente Figueiredo (number not specified) | 2,500,000 | Not reported in the study |
| 11 | 1 | 46,325,074.92 | R$/yr | 2008 | Aggregated over the number of households in Manaus (281,167) | 4,487 | Reported in the study |
| 12 | 1 | 389,811.84 | R$/yr | 2002 | Aggregated over the number of people living in the municipality Diamantino (18,457) | 763,000 | Not reported in the study |
| 13 | 1 | 5.78 | R$/visitor | 2015 | 50,000 visitors per year | 23,774 | Reported in the study |
| 14 | 1 | 613,844.1 | R$/yr | 2017 | 1,487 persons who live near the port of Camarán in the city Salvaterra | 10,800 | Reported in the study |
| 15 | 1 | 13 | USD/ha/yr | 2010 | Families living in the reserve Chico Mendes (number not specified) | 950,570 | Not reported in the study |
| 16 | 1 | 26,799,004.08 | R$/yr | 2008 | 200 thousand local residents in 13 municipalities | 43,400 | Reported in the study |
| 17 | 1 | 5.97 | R$/person/yr | 1997 | 1,877,015 residents living in the Federal District region, including the capital city Brasilia | 420,000,000 | The whole Brazilian Amazon |
| 17 | 2 | 5.14 | R$/person/yr | 1997 | 1,877,015 residents living in the Federal District region, including the capital city Brasilia | 89,500,000 | Great Carajas Program (GCP) |
| 17 | 3 | 4.38 | R$/person/yr | 1997 | 1,877,015 residents living in the Federal District region, including the capital city Brasilia | 330,500,000 | The whole Brazilian Amazon without the GCP |
| 18 | 1 | 337.82 | R$/ha/yr | 2017 | 94,289 people living in the protected area (PSA) in the river basin of Tangará da Serra | 5,417.7 | Reported in the study |
| 18 | 2 | 751.31 | R$/ha/yr | 2017 | 94,289 people living in the protected area (PSA) in the river basin of Tangará da Serra | 5,417.7 | Reported in the study |
| 19 | 1 | 1,294.25 | R$/hh/month | 2017 | 54 households living in the neighborhood of Buritis in the city Tangará da Serra | 5 | Not reported in the study |
| 20 | 1 | 1,255,593 | R$/year | 2015 | 34,000 visitors per year | 1.2 | Reported in the study |
| 21 | 1 | 2,085,018.47 | R$/year | 2012 | Aggregated over the number of visitors (9,590 derived from information in the study) | 429,600 | Reported in the study |
| 21 | 2 | 42,546.75 | R$/year | 2012 | Aggregated over the number of visitors (9,590 derived from information in the study) | 429,600 | Reported in the study |
| 22 | 1 | 1,977 | R$/year | 2007 | Area 1-aggregated across 25 families | 63.5 | Reported in the study |
| 22 | 2 | 2,635.2 | R$/year | 2007 | Area 2-aggregated across 30 families | 127.5 | Reported in the study |
| 23 | 1 | 23.52 | R$/inhabitant/month | 1995 | 72,500 inhabitants | 22,413,130 | Reported in the study |
| 24 | 1 | 174.87 | R$/hh/month | 2010 | 272 families | 424,430 | Reported in the study |
| 25 | 1 | 61.44 | USD/person/month | 2003 | 3.5 million inhabitants in the state of Amazonas | 157,074,570 | Not reported in the study |
| 25 | 2 | 74.33 | USD/person/month | 2003 | 3.5 million inhabitants in the state of Amazonas | 157,074,570 | Not reported in the study |
| 25 | 3 | 120.22 | USD/person/month | 2003 | 3.5 million inhabitants in the state of Amazonas | 157,074,570 | Not reported in the study |
| 26 | 1 | 29.22 | R$/person/month | 2006 | Local population of 46,285 persons | 104,010 | Reported in the study |
| 27 | 1 | 210.58 | R$/person/year | 2008 | Local population of 42,927 persons | 43,400 | Reported in the study |
| 28 | 1 | 2.12 | R$/scholarship holder/month | 2003 | 7,775 scholarship holders | 2,272,000 | Reported in the study |
| 29 | 1 | 4,073.84 | R$/ha | 2012 | Aggregated over 8,956 households living in the 20 surveyed villages | 20,597.5 | Reported in the study |
| 29 | 2 | 4,415.56 | R$/ha | 2012 | Aggregated over 8,956 households living in the 20 surveyed villages | 20,597.5 | Reported in the study |
| 30 | 1 | 215.66 | R$/person/year | 2014 | Local population: 130,660 persons | 72,200 | Reported in the study |
| 30 | 2 | 4,176.64 | R$/person/year | 2014 | Local population: 130,660 persons | 72,200 | Reported in the study |
| 31 | 1 | 9 | US$/ha | 2000 | Aggregated over hectares (population not specified) | 31,103,020 | Mato Grosso |
| 31 | 2 | 18 | US$/ha | 2000 | Aggregated over hectares (population not specified) | 420,000,000 | The whole Brazilian Amazon |
| 31 | 3 | 31.2 | US$/ha | 2000 | Aggregated over hectares (population not specified) | 420,000,000 | The whole Brazilian Amazon |
| 32 | 1 | 6.8 | R$/person/month | 2009 | Population living in the surveyed municipalities in the river basin (373,876) | 2,800,000 | Not reported in the study |
| 33 | 1 | 23,946,380 | R$/year | 2002 | Aggregated over 50 thousand visitors per year | 52 | Reported in the study |
| 34 | 1 | 17,573,973.87 | USD/year | 2004 | Aggregated over 159,998 households | 15,000,000 | Not reported in the study |
| 35 | 1 | 156,720 | R$/year | 2012 | Aggregated over 407,023 persons | 640,700 | Reported in the study |
| 36 | 1 | 5,750,000 | R$/year | 2009 | Aggregated over all local recreational fishermen (1,350) | 1,600,000 | Reported in the study |

^1^ Study numbers correspond to the numbering of studies in Table S1 in the Supporting Information.

**Table S4. Overview of available external socio-economic and environmental data**

|  | **Measurement units** | **Time scale** | **Spatial scale** | **Data source** |
| --- | --- | --- | --- | --- |
| **Socio-economic indicators** |  |  |  |  |
| Population density | Inhabitants/km^2^ | 2010 | Municipality | IBGE, Demographic Census 2010 |
| Share urban area | % | 2010 | Municipality | IBGE, Demographic Census 2010 |
| Monthly household income | R$/month | 2010 | Municipality | IBGE, Demographic Census 2010 |
| **Land use pressure indicators** |  |  |  |  |
| Share agricultural area | % | 1990-2011 | State | IBGE, 2012; Santos et al., 2013 |
| Cattle farming | Number of cattle heads/km^2^ | 1974-2009 | State | IBGE, Demographic Census 1940-2010 |
| Deforestation rate | % | 2000-2015 | State | INPE, 2015; Santos et al., 2013 |
| **Environmental indicators** |  |  |  |  |
| Share of forest area | % | 2000-2015 | Municipality | INPE, 2015 |
| Share of non-forest area | % | 2000-2015 | Municipality | INPE, 2015 |
| Share of water area | % | 2000-2015 | Municipality | INPE, 2015 |
| Share of protected areas | % | 2012 | State | IBGE, Demographic Census 2010; Santos et al., 2013 |

Explanatory notes:

IBGE: Instituto Brasileiro de Geografia e Estatística (Brazilian Institute for Geography and Statistics), <http://www.ibge.gov.br/home/>

INPE: Instituto Nacional de Pesquisas Espaciais (National Institute for Space Research), http://www.dpi.inpe.br/prodesdigital/prodesmunicipal.php

**Table S5. Results of the leave-one-out cross-validation procedure to estimate the predictive power of the estimated meta-regression models**

|  | **Relative RMSE**^1^  **(%)** | **Relative MAPE**^2^ **(%)** |
| --- | --- | --- |
| Mean value transfer all ecosystem services (n=61)^3^ | 85.2 | 68.9 |
| Model I (all ecosystem services, without valuation methods) | 70.8 | 56.0 |
| Model II (all ecosystem services, with valuation methods) | 70.3 | 50.7 |
|  |  |  |
| Mean value transfer all ecosystem services excluding outliers (n=53)^3^ | 78.8 | 68.6 |
| Model III (all ecosystem services, with valuation methods) | 66.7 | 48.2 |
|  |  |  |
| Mean value transfer habitat for species (n=26)^3^ | 83.5 | 70.3 |
| Model IV (habitat for species) | 76.2 | 56.1 |

^1^ RMSE: root mean squared error

^2^ MAPE: mean absolute prediction error

^3^ Predictive power without estimated meta-regression model based on sample average approximation.

**Figure S1. Distribution of value estimates across the Brazilian Amazon as a whole and its states**

**Figure S2. Scatter plot of value estimates in 2020 US dollars/ha/year across valuation methods**


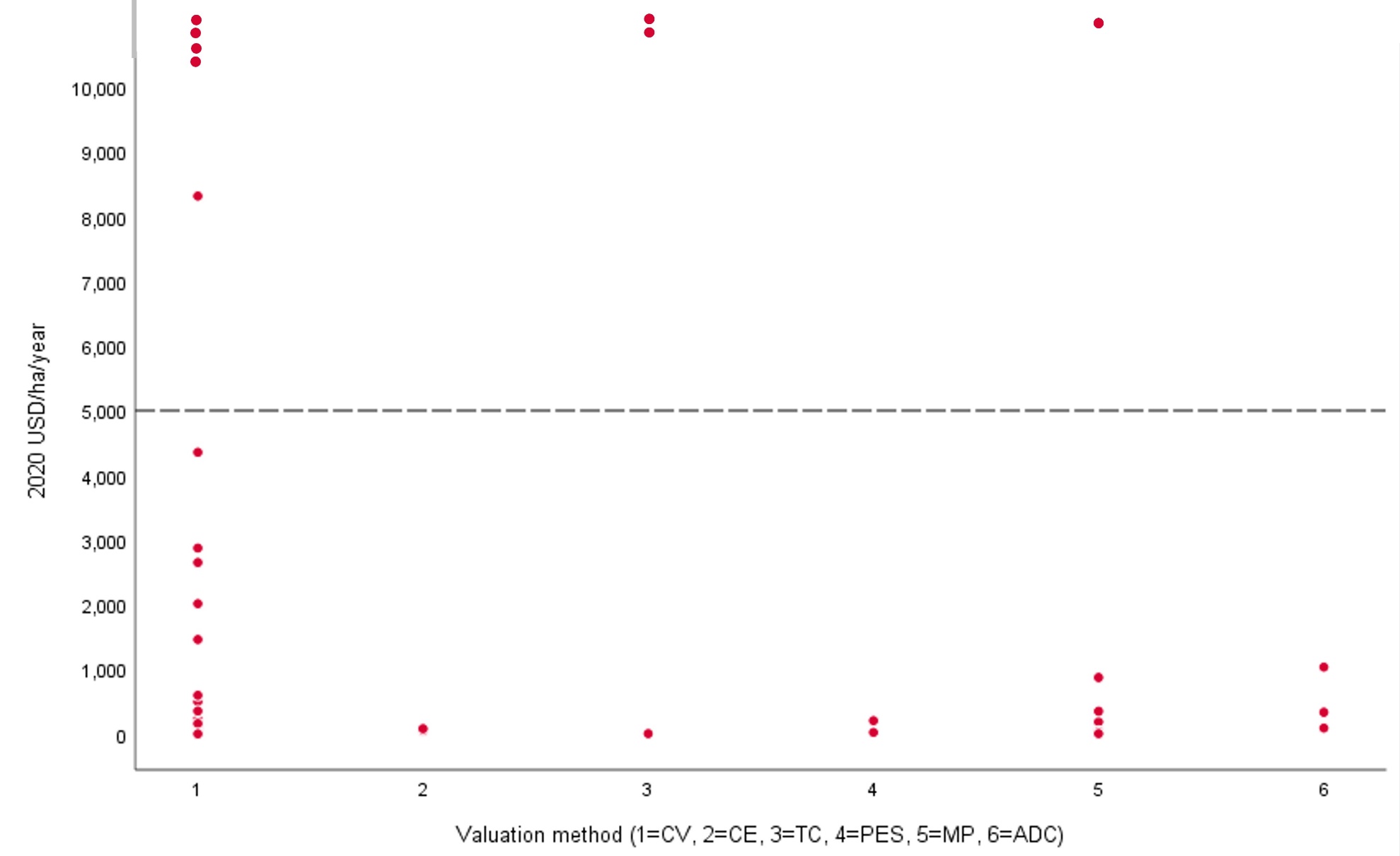


Note: outliers are located above the dashed horizontal line. CV: contingent valuation; CE: choice experiment; TC: travel costs; PES: payments for ecosystem services; MP: market prices; ADC: avoided damage costs.
